# Longitudinal characterization of maternal and infant salivary proteomic profiles across pregnancy and early infancy

**DOI:** 10.1186/s12884-026-09806-7

**Published:** 2026-08-14

**Authors:** Camila Lopes Crescente, Emily Freitas Lima, Juliana Mozer Sciani, Cristiane Duque, Vanessa Pardi, Ramiro Mendonça Murata, Thaís Manzano Parisotto

**Affiliations:** 1 Laboratory of Clinical and Molecular Microbiology, São Francisco University, Av. São Francisco de Assis, 218, Bragança Paulista - SP, 12916-900 Brazil; 2 Laboratory of Multidisciplinary Research, São Francisco University, Bragança Paulista - SP, Brazil; 3 Centro de Investigação Interdisciplinar em Saúde (CIIS), Universidade Católica Portuguesa (UCP), Viseu, Portugal; 4 Department of Foundational Sciences, ECU School of Dental Medicine, Greenville, NC USA

**Keywords:** Pregnant women, Infant, Saliva, Proteomics, Oral health

## Abstract

**Background:**

Pregnancy and infancy are critical stages for health intervention and physiological change. Although salivary proteomic shifts have been reported in pregnant women and infants, simultaneous mother-infant profiling—particularly using mass spectrometry—remains unexplored.

**Objective:**

To investigate the salivary proteomic profile in the mother-baby dyad, considering the third trimester of pregnancy.

**Materials and methods:**

Twenty pregnant women (PW) in the last trimester were selected and followed up. The PW were examined to assess caries experience (DMFT) and periodontal condition (CPI). Income, education, body mass index (BMI), and dietary practices were assessed through a questionnaire. Saliva samples were collected during pregnancy and postpartum (PP), as well as from the one-month infants (IF), and analyzed using mass spectrometry. Data were evaluated by descriptive statistics and bioinformatics.

**Results:**

PW in the study were from mid-level socioeconomic backgrounds with a high prevalence of caries (80%) and periodontal changes (60%). The average BMI was 28.37 kg/m^2^ in pre-pregnancy, indicating an overweight status, and the mean weight gain by the 3rd trimester was 7.23 kg. 35–40% of PW reported high consumption of carbohydrate. Proteomic analysis qualitatively identified 21, 27, and 25 proteins in PW, PP, and IF, respectively, with five proteins common to all groups. Key protein interactions were observed for salivary amylase (AMY1A, AMY1B, AMY1C), which are supposed to be linked to overweight. Gene Ontology (GO) enrichment analysis identified common genes and functional attributes across groups, with variation in expression intensity. Under GO molecular function, similarities were restricted to the PW and PP groups—specifically regarding alpha amylase activity and calcium, chloride, metal and antigen ion binding. Conversely, GO biological process analysis demonstrated that oligosaccharide metabolism was conserved across all groups (PW, PP, and IF).

**Conclusion:**

The salivary proteomic profiles of PW, PP, and IF shared several qualitatively identified proteins and showed notable similarities, suggesting candidates for further evaluation in maternal and neonatal health.

**Supplementary Information:**

The online version contains supplementary material available at https://doi.org/10.1186/s12884-026-09806-7.

## Introduction

Pregnancy and early childhood are periods of considerable physiological and behavioral changes, critical stages for establishing healthy habits, and unique opportunities for health-promoting interventions. Increased progesterone and estrogen in maternal circulation during pregnancy can lead to immune system modifications [1]. Metabolic changes can substantially increase food intake (quantity and frequency), particularly concerning carbohydrate consumption, which may be 15% higher than during the non-gestational period, favoring overweight or obesity [1].

Daily oral hygiene becomes difficult during pregnancy due to episodes of nausea and vomiting [2]. Hormonal changes and inadequate oral hygiene during pregnancy amplify the inflammatory response to dental biofilm, leading to gingival inflammation and an increased tooth decay, especially when combined with sugar consumption [2]. Inflammation of teeth-support tissues, pre-gestational overweight, and gestational obesity have been linked to systemic disturbances, including pre-eclampsia, premature birth, and low birth weight [3].

Prenatal dental care is crucial for establishing appropriate preventive and therapeutic strategies and assuring the mother’s good oral health regarding dental caries and periodontitis, for example [2]. More importantly, educational counseling during pregnancy directly impacts the prevention of early childhood caries (ECC) [4].

Saliva is a vital biological fluid and a source of biomarkers, due to its wide range of bioactive proteins and peptides [5]. Interestingly, its capacity to reflect serum characteristics related to systemic diseases has already been reported [5, 6]. As for its diagnostic potential and non-invasive assessment, analyzing salivary proteins is a broad research topic of increasing interest. Over 2,000 proteins and peptides have been detected in saliva, playing diverse roles in various biological processes within the oral cavity and the human body [5]. Advanced proteomic techniques, such as mass spectrometry, enable the identification of numerous biomarkers, which are valuable tools for early diagnosis, disease monitoring, and treatment [7–9].

Differences in the human salivary proteome have been highlighted in pregnant women [9], preterm newborns [10], and infants [11]. Although limited, salivary proteomics studies in pregnancy have identified potential biomarkers, such as elevated Annexin-1 levels in women with gingivitis [12] and Metallothionein 2 (MT2A) as a possible indicator of preterm birth [10], for example. Manconi et al. (2013) [11] used RP-HPLC–ESI–MS analysis to quantify proteins and peptides, revealing marked changes in the salivary proteome of children from birth to 48 months, and, except for statherin and histatin-3, major salivary proteins showed their lowest concentrations in the 0–6-month age group. Interestingly, recent literature has increasingly examined salivary cortisol and α-amylase as potential stress biomarkers [13]; nonetheless, findings in pregnant women did not yield significant results when compared to the postpartum period [14].

However, to our knowledge, no evidence is available to evaluate the salivary proteins of mothers and their early-life infants simultaneously, particularly using mass spectrometry. Our descriptive longitudinal study aims to understand potential changes in the proteomic profile of saliva, thereby elucidating the biological mechanisms that influence both oral and general health. Therefore, we aimed to investigate the salivary proteins in the mother-baby dyad, from the third trimester of pregnancy until the first month after the baby’s birth.

## Materials and methods

### Study design and population

Following the Declaration of Helsinki, this longitudinal observational study was conducted between November 2023 and July 2024, after obtaining ethical approval (Ethics Committee of the University São Francisco - protocol number: 65319322.2.0000.5514) and receiving written consent.

This study conveniently recruited pregnant women and their infants from the largest Gynecology and Obstetrics Clinic of Extrema, Brazil. Extrema city is located in the south of Minas Gerais State, has ≅ 36.951 inhabitants, presents a human developmental index of 0.732, and optimally fluoridated tap water (0.69 mg F/L).

This study was part of a larger investigation involving 104 participants. Healthy pregnant women (PW) in their third trimester (between 28 and 41 weeks of gestation) who were receiving prenatal care at the participating clinic were eligible for inclusion. Eligible participants were required to provide an adequate saliva sample (flow rate > 0.3 ml/min [15]) and have no history of hypertension, gestational diabetes mellitus, or orthodontic appliance use at the time of enrollment.

Exclusion criteria relied on use of antibiotics, corticosteroids, or antihypertensive medications during the study period or within the month preceding enrollment, and the presence of neuromotor or communication impairments or psychiatric disorders.

Among the 45 women who completed the pregnancy assessment, 11 declined further participation after delivery and 14 were lost in the follow-up, due to inability to re-establish contact, relocation, or missing postpartum visits (Figure S1). Based on baseline data women who completed the follow-up did not differ meaningfully from those who did not. Only mother–infant dyads with complete maternal follow-up and infant saliva collection at one month postpartum were included in the final longitudinal analyses. A total of 20 mother–infant dyads fulfilled these requirements and were included in this study (40 participants). Individuals were classified into three groups: pregnant women (PW group), postpartum women (PP group—the same participants evaluated after delivery), and infants (IF group).

### Assessment of pregnant women’s oral health and dietary practices

The oral exam was conducted in the clinic’s waiting room. The presence or absence of dental biofilm was assessed by visually examining the buccal surfaces of maxillary incisors. After that, PW had their teeth cleaned and dried with gauze (Medi House, São Paulo-SP, Brazil), and the diagnosis of dental caries (decayed, missing, and filled surfaces and teeth - DMFS/DMFT index) and periodontal evaluation (Community Periodontal Index - CPI) was performed according to the World Health Organization (WHO) criteria [16]. The diagnosis was conducted using artificial light (Sfl5540, Wat Nichia Philips, Brazil), a mirror, and a ball-ended dental probe (Indusbello, Londrina- PR, Brazil). Clinical examination was performed by one dentist, previously calibrated by a gold standard examiner, who received all the theoretical and practical instructions regarding the criteria to be used. About 10% of the sample was re-examined during the calibration process with a 1-week interval. A Kappa statistic was calculated to ensure homogeneous intra-examiner assessment, indicating excellent agreement, with a value of 0.86. Pregnant women who needed dental treatment were referred for clinical care.

Maternal dietary practices during pregnancy were assessed using a structured and simplified questionnaire recommended by the WHO to determine the frequency of consumption of specific food groups among adults [16]. Socioeconomic questions were added to the questionnaire (“What is your family income?” and “What is your education level?“). In addition, the initial and final weights of the pregnant women were collected from medical records, and the body mass index was calculated according to the WHO guidelines [17]. After the baby was born, information regarding the baby’s weight and height was assessed from the baby’s information card. Information on the type of breastfeeding (exclusive, formula, or mixed) was obtained through direct interviews with the mothers.

### Saliva sample collection

Saliva from the PW was collected during the dental examination, in the morning, without any instructions regarding toothbrushing, as the presence/absence of dental biofilm was being analyzed. Postpartum women were instructed to refrain from eating or brushing their teeth for 1 h prior to saliva collection and to avoid feeding their babies during the same period. This standardized interval was adopted to minimize the potential influence of recent feeding on the infant salivary proteome. Saliva collection from the postpartum women and their infants was performed in the morning period when they completed one month of life, corresponding to a range of 30–35 days.

At least 500 µl of unstimulated mothers’ saliva were collected by passive drooling (3 min) in plastic cups and transferred to microcentrifuge tubes using sterilized disposable syringes. The babies’ saliva was collected using SalivaBio’s Infant Swab (SIS) Method (Salimetrics, USA). It allows an easy, clean, and less invasive collection, carried out in two steps: (1) The roll was inserted into the baby’s mouth until it was soaked with saliva; (2) The roll was removed and inserted into the swab storage plastic tube. Each infant sample was collected individually by gently placing the swab into the oral cavity until it became completely saturated with saliva, without a predetermined collection time. All infant samples yielded sufficient saliva for proteomic analysis using the same collection procedure, and no samples were excluded or recollected because of insufficient material. Saliva was kept refrigerated during the entire collection period. All saliva samples were transported under refrigeration to the Laboratory of Clinical and Molecular Microbiology. The infant`s saliva samples obtained with SIS Method (Salimetrics, USA) were recovered by centrifugation at 5,500 rpm for 10 min under refrigeration, and then the supernatant was transferred to 1.5 ml microtubes. Approximately 300 µL of saliva was recovered from each SIS following centrifugation. All samples were frozen at -80 °C.

### Saliva preparation for protein mass spectrometry analysis

In the Clinical and Molecular Microbiology Laboratory, samples were thawed, and the preparation was carried out on ice when applicable. First, sample concentrations were normalized based on total protein content determined by the Bradford assay (BioRad, Hercules, CA, USA) and all samples were adjusted to the same protein mass (80 µg). Then, fifty microliters of 8 M urea solution were added to each 100 µl of normalized saliva sample. After vortexing, the samples were heated in a dry bath at 80 °C for 15 min to denature proteins by disrupting hydrogen bonds, followed by centrifugation to remove potential cellular debris. Five microliters of 100 mM dithiothreitol (DTT) were added, the samples were vortexed again and incubated at 60 °C for 30 min. After cooling to room temperature, the samples were centrifuged again, and 5 µl of 300 mM iodoacetamide was added. Due to the sensitivity of iodoacetamide to light, samples were kept in a dark box at room temperature for 30 min. Then, 200 µl of 50 mM Tris-HCl (pH 8) were added to dilute urea to 0.5 M and 20 µl of trypsin solution (40 ng/µL in 50 mM NH₄HCO₃) were added, to achieve 1:100 enzyme-protein ratio. Samples were vortexed and incubated overnight at 37 °C for digestion. To stop the enzymatic reaction, 20 µL of 5% trifluoroacetic acid (TFA) was added. Afterward, the samples were frozen at -80 °C, lyophilized for 2 h, and stored at -20 °C. Samples were diluted in ultrapure water containing 0.1% formic acid for mass spectrometry analysis, which was performed within 1 h [18].

### Salivary proteins for mass spectrometry analysis

Mass spectrometry is an advanced analytical technique that identifies molecules based on their mass-to-charge ratio (m/z). For protein analysis, 10 µl aliquots of prepared saliva samples were applied to a C18 column (130Å, 1.7 μm, 2.1 mm x 50 mm, ACQUITY UPLC BEH, Waters Co). The eluted peptides were automatically injected into a Q-ToF Xevo G2-XS mass spectrometer (Waters Co., USA). A clean-up step was achieved by 2 min of wash with 0% B to remove salt and no retained compounds (no addressed to the mass spectrometry), and then a linear gradient (1–40% B; A = 0.1% formic acid; B = 90% acetonitrile in 0.1% formic acid) at constant flow of 0.2 mL/min for 90 min, at 30 °C. Spectral data were collected in positive ionization mode (200–1800 m/z) with a resolution of 40,000 FWHM at a mass-to-charge ratio (m/z) of 500, with MS/MS acquisition automatically, with a 10 more intense ions. These ions were fragmented with collision energy according to their m/z, ranging from 10 to 50 V. Parameters were set at 3 kV capillary voltage, 150 °C source temperature, 350 °C desolvation temperature, and 600 L/h desolvation gas flow. Argon collision energy was used for MS/MS analysis, with intensity adjusted based on peptide charge. MassLinx 4.2 software was used for instrument control and data acquisition [18]. All saliva samples collected during pregnancy, postpartum, and infancy were processed and analyzed individually, and no sample pooling was performed.

### Statistical analysis and data visualization

Population characteristics, including maternal age, BMI, gestational weight gain, periodontal/dental caries index, infant weight and length at birth/1 month, and household income, were assessed using descriptive statistics. The proteome data were analyzed qualitatively using Peaks Studio V.7.0 software (Bioinformatics Solutions Inc., Waterloo, ON, Canada) alongside descriptive statistical methods. The proteins that we identified agreed with the Peaks Studio software statistical parameters. For protein identification, raw mass spectrometry files were imported into Peaks Studio, and identification was performed using the International Protein Index (IPI) and a custom database with proteins deposited in UniProt (www.uniprot.org) retrieved using terms “secreted proteins” and “human”. Key search parameters included MS and MS/MS tolerance set to 2 ppm, fixed modification for carbamylation, variable modifications for carbamidomethylation, deamination (at N and Q), N-terminal acetylation, and oxidation at M, a maximum of three missed cleavages, considering only tryptic cleavage, and up to three variable post-transcriptional modifications (PTMs) per peptide. A false discovery rate (FDR) of ≤ 0.5% and ALC score > 50% were considered. Proteins with a -10lgP ≥ 20 and at least one unique peptide were included in the study.

**Fig. 1:**
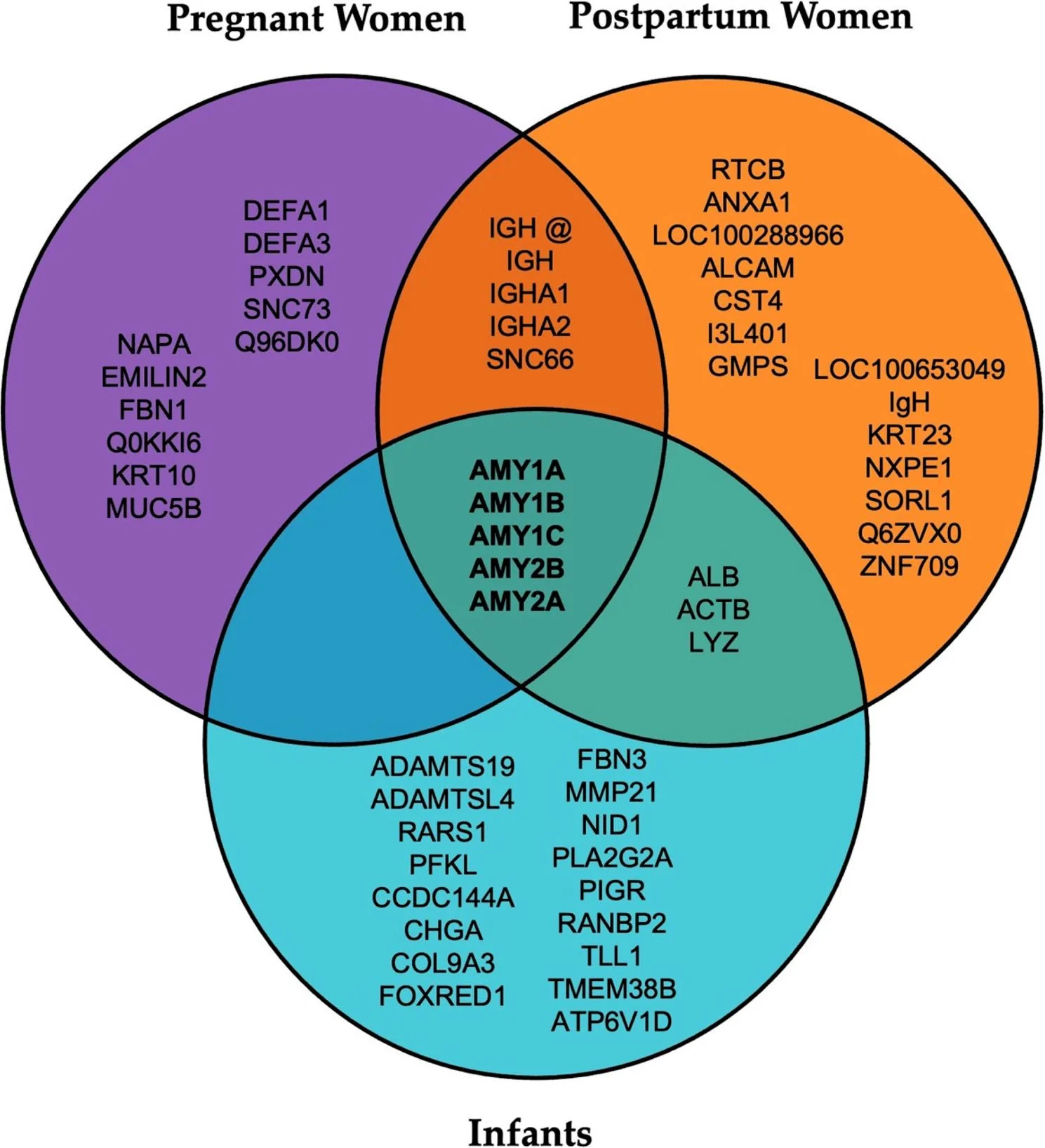
Distinct and shared proteomic signatures across pregnant women, postpartum women, and infants. Venn diagram illustrating the distribution of identified proteins among three groups: Pregnant Women (PW), Postpartum Women (PP), and Infants (IF). Each circle represents the proteome profile of a specific group, highlighting proteins that are exclusively detected in one group (outer regions) and those shared among two or all three groups (overlapping regions). Notably, five amylase isoforms (AMY1A, AMY1B, AMY1C, AMY2A, AMY2B) were the unique proteins shared across all groups

**Fig. 2:**
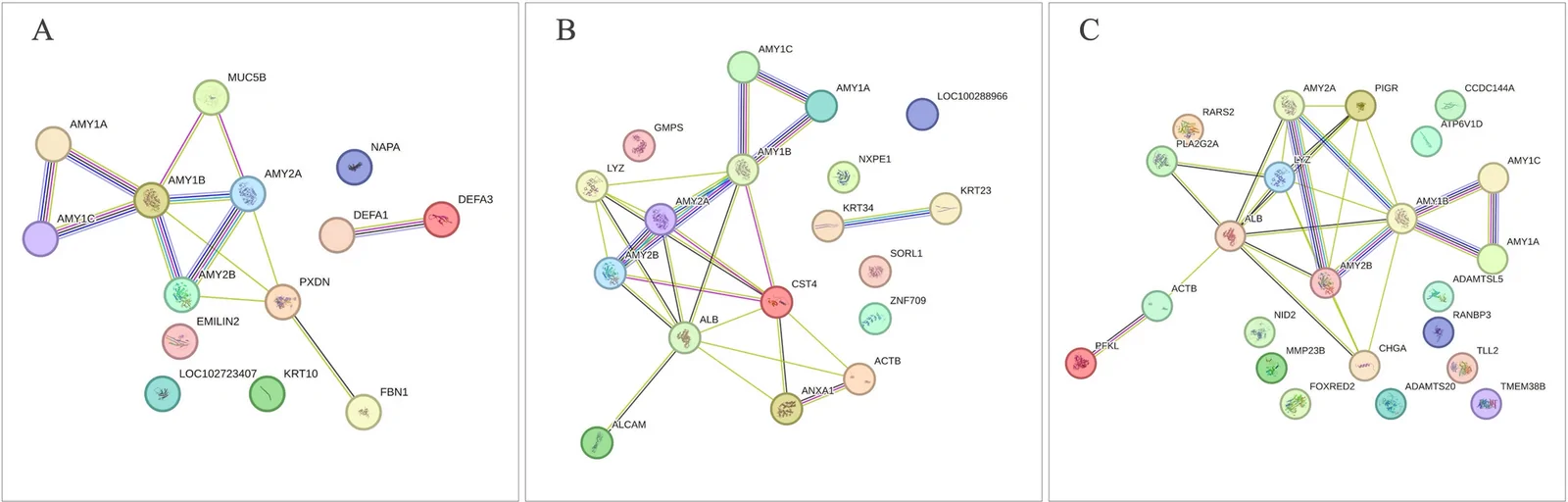
STRING biological pathway according to the studied groups. Protein-protein interaction networks of the identified proteins in (**A**) pregnant women, (**B**) postpartum women, and (**C**) infants. Each node represents a protein, and edges indicate known or predicted interactions. Network statistics: (A) number of nodes: 14; number of edges: 13; average node degree: 1.86; avg. local clustering coefficient: 0.607. (B) number of nodes: 18; number of edges: 25; average node degree: 2.78; avg. local clustering coefficient: 0.645. (C) number of nodes: 23; the number of edges: 26; average node degree: 2.26; avg. local clustering coefficient: 0.414

**Fig. 3:**
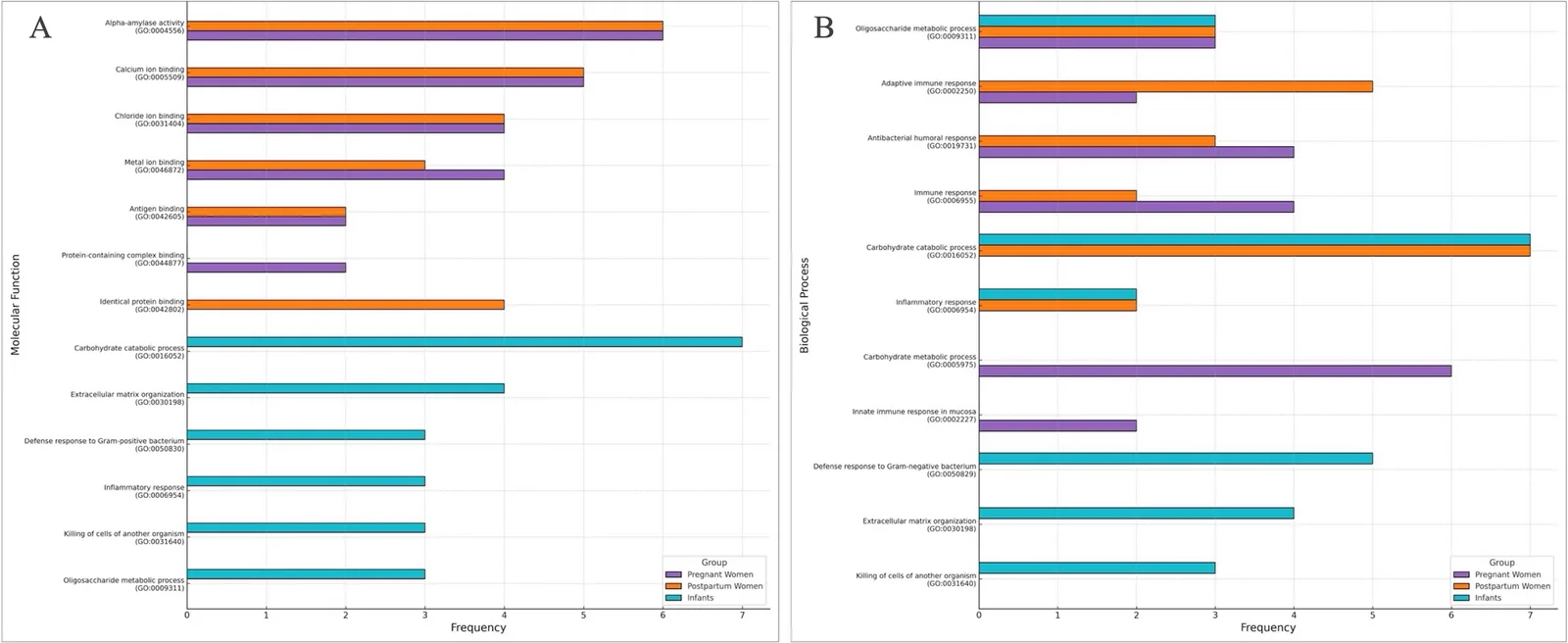
**A** Gene-Ontology (GO) considering the identified proteins’ molecular function. Gene Ontology (GO) analysis reveals the functional categorization of proteins found in pregnant women (purple), postpartum women (orange), and infants (blue). Bars represent the frequency of proteins associated with each molecular function, highlighting both shared and group-specific roles. **B** Gene-Ontology (GO) considering the identified proteins’ biological process. Gene Ontology (GO) analysis reveals the process categorization of proteins found in pregnant women (purple), postpartum women (orange), and infants (blue). Bars represent the frequency of proteins associated with each biological process, highlighting both shared and group-specific roles

Identified proteins were examined using Venn Diagrams for comparative expression with CNB-CSIC Venny 4 (an online tool) (Fig. 1). Gene ontology was performed using the UniProt database (Fig. 3-A and -B), and biological pathway enrichment (Fig. 2) was analyzed via the String program (version 11.5, online tool).

## Results

### Participants’ characteristics

The characteristics of pregnant women, including age, body mass index, dental caries index, income, education, and dietary practices, are presented in Table 1. Pregnant women were overweight and approximately eight teeth were affected by caries lesions per woman. The major component of the dental caries index was dental restorations (69%). Based on the CPI index, 40% of the pregnant women had all sextants in healthy conditions. Considering the worst condition identified in at least one sextant, bleeding was observed in 20% of the pregnant women, 30% had dental calculus, and 10% had periodontal pockets of 4–5 mm or sextant exclusion due to the absence of teeth (less than two).

**Table 1:** Populations’ characteristics

	Mean	Standard Deviation	Median	Interquartile Range
PW Age (years)	29.63	± 8.31	29	± 8.50
Pre-pregnancy *BMI* (Kg/m^2^)	28.37	± 6.29	28.26	± 5.92
Weight gain during pregnancy (Kg)	7.23	± 4.34	7.20	± 5.50
PW Dental Caries index *(DMFT/DMFS)*	7.94 / 13.63	7.30 / 15.59	8 / 10	6.50 / 6.50
Infant’s birth weight and length (Kg/cm)	3.02 / 48.42	0.49 / 1.39	3 / 48	0.58 /2.25
Infant’s weight and length after 1 month (Kg/cm)	4.03 / 53.70	0.85 / 2.62	4.30 / 53	1.03 / 4.25
Income (USD)	695	522	519	329
		**Highest education degree**		
		**Number**	**%**	
Middle school		2	10	
High school		13	65	
College		5	25	
		**Consumption more than once a week**		
		**Number**	**%**	
Cookies and Cakes		8	40	
Sweets and candies		4	20	
Soft drinks or lemonade		4	20	
Tea with sugar		7	35	
Coffee with sugar		8	40	

*BMI *Body Mass Index*, DMFT *decayed, missing, or filled teeth (permanent),* DMFS *decayed, missing, or filled surface

Regarding feeding practices in the first month postpartum, 60% of the babies were exclusively breastfed, 30% combined breastfeeding with formula, and 10% were exclusively formula-fed. Although 90% of pregnant women report consuming fresh fruit at least once a week, the frequency of daily consumption was low (35%).

### Proteomic profile

Proteomic analysis identified 55 distinct proteins (Table S1 - Supplementary Table 1) in the saliva samples of the studied population, and Fig. 1 illustrates the overlapping proteins among the groups. For the qualitative proteomic analyses, a protein was considered present in a study group if it was identified in at least one individual sample from that group. Accordingly, group-specific and shared proteins shown in the Venn diagram were defined based on the presence or absence of protein identification within each group, irrespective of the number of participants in whom the protein was detected. Eleven, 14 and 17 proteins were detected only in groups PW, PP, and IF, respectively. Five proteins were obligatorily shared among all groups: salivary amylases (AMY1A, AMY1B, AMY1C) and pancreatic alpha-amylases (AMY2A, AMY2B). Three other proteins (Albumin, Actin cytoplasmic 1, and Lysozyme C) were commonly identified in both the PP and the IF groups. Only five proteins (IGH@ protein, Immunoglobulin heavy chain variant, Immunoglobulin heavy constant alpha 1, Immunoglobulin heavy constant alpha 2, and SNC66 protein) were shared between the PW and PP groups. No protein was concomitantly found in the PW and the IF groups.

Biological Pathways (Fig. 2) analyses show the interaction maps of proteins identified in each group, suggesting the molecular networks specific to pregnant women, postpartum women, and infants. In these networks, nodes represent individual proteins, while the connecting lines (edges) denote established or predicted possible functional associations. Generally, distinct protein compositions and interactions were found among the PW, PP, and IF groups. However, a notable pattern of solid interactions between the same protein’s genes deserves to be highlighted: amylases (AMY1A, AMY1B, AMY1C, AMY2A, and AMY2B).

Figures 3 display the Gene Ontology analyses (obtained from the UniProt database) according to molecular function (A) and biological process (B). Gene ontology (GO) is a taxonomy that statistically determines the sets of genes involved in the same biological processes, functions, or localizations.

Regarding molecular function (GOMF), no one was common to all three groups. Still, several were shared between the pregnant women (PW) and postpartum (PP) groups with the same frequency (alpha-amylase activity, calcium ion binding, chloride ion binding, antigen binding) as shown by the horizontal bars in the gene ontology (Fig. 3-A). In contrast, the most prevalent suggested molecular functions in the infant (IF) group were carbohydrate catabolic process. Oligosaccharide metabolic process, another function that may be related to carbohydrate digestion, was also identified in this group. Interestingly, we also observed possible functions associated with innate immunity, including extracellular matrix organization, defense response to Gram-positive bacteria, inflammatory response, and killing of cells from other organisms (Fig. 3-A).

Analysis of the biological process (GOBP) protein patterns revealed differences among all studied groups, with only one suggested process, the oligosaccharide metabolic process, shared by all three groups, as indicated by the horizontal bars in the Gene Ontology (Fig. 3-B). The carbohydrate catabolic process seemed to be the most prevalent biological process. It appeared only for PP and IF, while the most prevalent biological process for pregnancy was the carbohydrate metabolic process. The inflammatory response was shared between the PW and IF groups. Furthermore, in the IF group, possible processes related to immune response (defense response to Gram-negative bacteria, killing of cells of another organism) deserve highlighting.

## Discussion

It is well known that saliva is a rich biological fluid and a potential source of biomarkers. The results of the present study highlight remarkable interrelationships in the proteomic profile of pregnant women (PW), postpartum women (PP), and their infants (IF). Unstimulated salivary samples seemed to be a good choice for proteomic analysis [11, 19]. In the PW, we identified 21 different proteins, similar to the findings of Balan et al., 2021 [20] (28 proteins) and comparable to pregnant women in other pregnancy periods (47 and 18 proteins for early and mid-gestational stages, respectively) reported by Dey et al., 2020 [21]. Interestingly, these authors [21] pointed to an inverse relationship between the number of proteins and gestational age, i.e., the higher the gestational period, the lower the number of proteins found. Concerning the PP and IF groups, age-related variations in the salivary proteome have been reported in preterm newborns and infants [11, 22], underscoring the influence of developmental stage on protein expression; however, these findings were obtained using different techniques.

Interestingly, in all groups of the present investigation, amylase 1B (AMY1B), a salivary isoenzyme synthesized by all three major glands, with the parotid gland being the predominant source, was identified. Previous research identified five alpha-amylase isoforms in obese women during late pregnancy, noting a ten-fold upregulation in those with concurrent periodontitis [23]. Furthermore, within a similar cohort, the specific presence of AMY1B was associated with severe gingival bleeding on probing (> 50%) [24]. AMY1B is involved in carbohydrate metabolism and influences appetite and weight regulation [25]. In general, amylases are secreted proteins capable of hydrolyzing 1,4-alpha-glucosidic bonds in oligosaccharides and polysaccharides, thereby catalyzing the initial step in the digestion of starch and glycogen. Additionally, our study identified pancreatic amylase, represented by the gene codes AMY2B and AMY2A, which acts in the small intestine [25]. In line with the above findings, the women’s initial BMI before pregnancy, indicated that they were overweight. In the third trimester, the mean weight gain indicated that the pregnant woman persisted in the overweight category. Pregnancy weight gain is linked to eating habits [26]. It was already reported that gestational weight gain increased by 1.33 kg for each 1-percentage-point rise in the proportion of dietary energy derived from ultra-processed foods [26]. Higher consumption of saturated fats and ultra-processed foods contributes also to greater postpartum weight retention [27]. Of interest, 35–40% of the pregnant women in our study reported consuming large quantities of sweet cookies and cakes, as well as sugar-sweetened tea and coffee. These foods are rich in sucrose and contain empty calories, contributing to increased weight gain during pregnancy and promoting unhealthy conditions in the oral cavity, such as caries lesions and gingivitis (precursor to periodontitis), due to biofilm accumulation [28]. High gestational weight gain has been linked to an elevated risk of dental caries even in their offspring [29]. Additionally, diet has a direct impact on the salivary protein composition [30].

Salivary amylases (AMY1A, AMY1B, AMY1C) and pancreatic alpha-amylases (AMY2A, AMY2B) form an interesting relationship that can be observed in the PW, PP, and IF groups, as shown in the STRING biological pathway, maybe due to the carbohydrate-rich dietary profile, including lactose. The interplay between dietary carbohydrate intake and AMY1 polymorphisms affected the incidence of type 2 diabetes in Korean women [31]. This dietary link is further supported by a fish model, where increased carbohydrate consumption triggered a significant rise in amylase levels [32]. Even though our study was a qualitative proteomic study, enabling the identification of some proteins using a single unique peptide, it might provide a first insight into how protein profiles associated with overweight appear in pregnant women, mothers, and their babies. Although the infant’s birth weights were average (higher than 2.5), and most of them were breastfed, their nutritional status warrants a follow-up investigation.

A broad view of the proteomic profile was evident in the Gene Ontology of salivary proteins. Gene Ontology is represented by the letters “GO” and is followed by a number, in a computer-readable format. It consists of sets of genes connected to functional molecules, such as proteins, which are statistically classified basically according to biological processes (GOBP) and molecular function (GOMF), which were included in the present study. GOMF analysis reveals similarities in terms of composition between the pregnant women (PW) and postpartum (PP) groups. Alpha-amylase activity (GOMF) was the most prominent in both groups, suggesting the role of amylases in these biological profiles. Calcium, chloride, and metal ion binding were the most prevalent molecular functions observed among these groups, according to GO analysis.

Calcium ion binding was the second most prevalent molecular function in PW and PP groups (GOMF). It is connected to calcium ion storage activity. Calcium ion plays a crucial role in salivary electrolyte dynamics and are essential for maintaining the integrity of dental tissues. It actively remineralizes carious lesions and promotes the supersaturation of minerals in saliva, thereby strategically minimizing the damage caused by saccharolytic bacteria [33]. Considering that the PW and PP groups involved the same individuals at distinct stages and that these women exhibited a high prevalence of caries lesions and a significant presence of visible biofilm, it might be inferred that essential changes in calcium ion bioavailability are occurring in the oral environment, favoring the expression of calcium-binding proteins. Interestingly, it has already been demonstrated that a more disorganized structure of the biofilm (immature) provides a significant absorption of calcium from saliva to the tooth-enamel interface, reducing its bioavailability in saliva, while a more organized biofilm (mature) does not, allowing more calcium availability in saliva [34]. Interestingly, in a study involving Brazilian pregnant women with obesity and periodontitis, a calcium binding protein (protein S100-A8) was found to be up-regulated in the saliva, with functions involved in inflammatory mechanisms [23].

Chloride ion binding (GOMF) is the third most prevalent molecular function shared by the PP and PW groups, referring to the ability of a protein to interact selectively with chloride ions (Cl). This interaction is crucial to many biological processes, including osmotic balance and the transport of ions. Most chloride anions are free, but some bind to proteins, influencing their spatial structure and transformations. Its functions include the regulation of enzyme activities and the prevention of infection [35]. The chloride ion binding (GOMF) connects one of the most essential transport and storage proteins in human blood plasma, albumin, to the oxygen-carrying protein in erythrocytes, hemoglobin [35]. Processes linked with infection prevention and good oxygen carriage are vital to human health.

Interestingly, metal ion and antigen binding (GOMF) are the last molecular functions common to PW and PP groups. While antigen binding is equivalent in the two groups, metal ion binding is more intense in the PW group. Metal ion binding is the ability of proteins to bind to metal ions in general (calcium, zinc, iron, copper), and antigen-binding molecules can bind to foreign antigens. This makes sense since binding to metal ions helps regulate enzymatic activity/defense against microorganisms [36], and binding to antigens induces or regulates the immune response. Bacteria can adhere to salivary proteins adsorbed on the tooth’s surface or to free salivary components, becoming agglutinated and removed from the oral cavity [36] by swallowing or coughing, thereby preventing oral colonization. This mechanism is crucial in protecting against systemic infection [37, 38].

Concerning the infant (IF) group molecular functions and biological processes, carbohydrate catabolic processes (GOMF, GOBP) predominated, and oligosaccharide metabolic processes were also notable. In the early life, digestible carbohydrates are primarily consumed as lactose, which brush-border lactase in the small intestine hydrolyzes into glucose and galactose for absorption via sodium-glucose cotransporters [39, 40]. In contrast, breast milk oligosaccharides are non-digestible and pass intact into the large intestine. There, they act as prebiotics to selectively stimulate beneficial microbiota, such as *Bifidobacterium*, ultimately promoting colonic health, modulating immune function, and lowering susceptibility to infection [40]. Thus, the fact that carbohydrate catabolic processes and oligosaccharide metabolic processes (assessed by GO protein analysis) were expressive in the present study may indicate a salivary profile adapted to sugar metabolism; following a milk-based diet, particularly breastmilk, consumed by 60% of the infants. These metabolic pathways are crucial during the neonatal period, as they support energy production and influence immune development and early microbiota composition [41].

Other protein`s molecular functions and biological processes, such as extracellular matrix organization, killing of cells from another organism, and defense responses to bacteria, deserve highlighting in the infant group, all of which are associated with innate immunity. This might align with the early postnatal period, characterized by immune immaturity and a heavy reliance on maternal support, particularly through breast milk, which is rich in antibodies, cytokines, and oligosaccharides (HMOs) [41]. According to Hibel et al. [42], salivary secretory IgA levels in one-month-old infants reflect maternal levels but decline sharply (by 70%) at 3 and 6 months before stabilizing. Enriching the immune-related protein pathways in infant saliva might reflect the transition from maternal to self-derived immunity, corroborating a gradual maturation of the infant’s mucosal immune system.

Still, concerning GO protein analysis, the carbohydrate catabolic process was the most prevalent biological process found in the IF and PP groups, followed by the carbohydrate metabolic process, observed in the PW group (GOBP). These findings may be linked to the high carbohydrate intake reported by the pregnant women in our study. Such dietary habits during pregnancy might influence maternal metabolism and shape the biochemical and microbial environment to which the infant is exposed. The molecular echoes of mothers’ feeding practices, possibly reflected in the infant salivary proteome, suggest a potential vertical correlation, where maternal nutritional behavior maybe could modulate early-life metabolic signatures and may impact early metabolic programming [43]. This observation supports that maternal-infant biological relationships are beyond genetics.

Among the biological processes (GOBP) identified, the oligosaccharide was the only one present in all three groups (PW, PP, and IF), perhaps supporting a critical link between maternal and infant physiology. Notably, variations in the concentration of human milk oligosaccharides (HMOs) [43] have been associated with maternal obesity. In a previous study, maternal BMI (one month postpartum) also correlated with the abundance of specific HMOs [44], which are known as key prebiotic agents. Therefore, the shared oligosaccharide metabolic process in the maternal-infant dyad suggests that maternal metabolic status may influence the proteomic profile, an insight that warrants further investigation.

The present longitudinal study, involving mothers and very young babies, has several strengths, including its ability to track multiple variables, which provides a comprehensive view of early development. On the other hand, the investigation has limitations. This was a small convenience sample, which may limit the generalizability of the findings. The pregnant women were from mid-level socioeconomic backgrounds, as indicated by their income and education levels. Therefore, future studies including participants with more diverse socioeconomic backgrounds, feeding practices, and oral health conditions, together with more detailed periodontal assessments, may provide additional insights. In addition, the proteomic analysis was primarily descriptive, and individual-level protein quantification and association analyses were not performed. Measuring/quantifying individual protein levels would strengthen the clinical relevance of these findings. Maternal saliva was collected by passive drooling, whereas infant saliva used a swab-based method. Although this approach was necessary because of the infants’ age, differences in collection procedures may have influenced protein recovery and should be considered when comparing maternal and infant proteomic profiles. While mothers were instructed not to feed their infants for at least one hour before saliva collection, the potential influence of recent feeding on the infant salivary proteome cannot be completely excluded. Furthermore, protein identification was based on qualitative detection criteria, including proteins supported by at least a single peptide, which may have increased the likelihood of low-confidence identifications for some proteins. As this is a preliminary study, further research with larger cohorts and mechanistic approaches is required to clarify the role of salivary protein dynamics in the metabolic health of the mother–infant dyad.

In conclusion, this longitudinal salivary proteomics study reveals qualitative similarities in the proteomic profiles among overweight pregnant women/mothers, and their infants, especially considering salivary alpha-amylase protein. While exploratory, these findings establish a foundation for future quantitative studies, providing priority targets for validation in larger, standardized maternal-neonatal cohorts.

## Supplementary Information

Supplementary Material 1.

## Data Availability

All data are available in the Repositories ProteomeX- change\_PXD063678 and jPOST\_JPST003789, as well as in Supplementary Table 1.
