# Supplementary material for: Longitudinal characterization of maternal and infant salivary proteomic profiles across pregnancy and early infancy
Source: BMC Pregnancy Childbirth. 2026 Aug 14;26:1309. doi: 10.1186/s12884-026-09806-7 (PMC13753420; doi:10.1186/s12884-026-09806-7)
Supplement: Supplementary file 1 [file 12884_2026_9806_MOESM1_ESM.docx]

# Supplementary File

# Figure S1 - Supplementary Figure 1

**Figure S1.** Flow chart of the sample selection.

# Table S1 - Supplementary Table 1

**Table S1.** Details of the proteins identified in the study, including UniProt entry terms, oficial protein names, gene symbols, and their respective experimental groups: pregnant women (PW group), postpartum women (PP group) and infants (IF group).

| **UniProt access number** | **Entry Names** | **Protein Names** | **Gene symbols** | **Groups** | | |  |
| --- | --- | --- | --- | --- | --- | --- | --- |
| [**B4DXF1**](https://www.uniprot.org/uniprotkb/B4DXF1/entry) | B4DXF1_HUMAN | 3'-phosphate/5'-hydroxy nucleic acid ligase | RTCB |  | PP |  | |
| **Q8TE59** | ATS19_HUMAN | A disintegrin and metalloproteinase with thrombospondin motifs 19 | ADAMTS19 |  |  | IF | |
| **Q6UY14** | ATL4_HUMAN | ADAMTS-like protein 4 | ADAMTSL4 |  |  | IF | |
| **P02768** | ALBU_HUMAN | Albumin | ALB |  | PP | IF | |
| **P0DUB6** | AMY1A_HUMAN | Alpha-amylase 1A | AMY1A | PW | PP | IF | |
| **P0DTE7** | AMY1B_HUMAN | Alpha-amylase 1B | AMY1B | PW | PP | IF | |
| **P0DTE8** | AMY1C_HUMAN | Alpha-amylase 1C | AMY1C | PW | PP | IF | |
| **P19961** | AMY2B_HUMAN | Alpha-amylase 2B | AMY2B | PW | PP | IF | |
| **P54920** | SNAA_HUMAN | Alpha-soluble NSF attachment protein | NAPA | PW |  |  | |
| **P04083** | ANXA1_HUMAN | Annexin A1 | ANXA1 |  | PP |  | |
| **P54136** | SYRC_HUMAN | Arginine--tRNA ligase | RARS1 |  |  | IF | |
| **P17858** | PFKAL_HUMAN | ATP-dependent 6-phosphofructokinase, liver type | PFKL |  |  | IF | |
| **P60709** | ACTB_HUMAN | Beta-actin | ACTB |  | PP | IF | |
| **A2RUR9** | C144A_HUMAN | CCDC144C-like coiled-coil domain-containing protein | CCDC144A |  |  | IF | |
| **A0AAG2SVG9** | A0AAG2SVG9_HUMAN | CCDC144C-like coiled-coil domain-containing protein | LOC100288966 |  | PP |  | |
| **Q13740** | CD166_HUMAN | CD166 antigen | ALCAM |  | PP |  | |
| **P10645** | CMGA_HUMAN | Chromogranin-A | CHGA |  |  | IF | |
| **Q14050** | CO9A3_HUMAN | Collagen alpha-3(IX) chain | COL9A3 |  |  | IF | |
| **P01036** | CYTS_HUMAN | Cystatin-S | CST4 |  | PP |  | |
| **Q9BXX0** | EMIL2_HUMAN | EMILIN-2 | EMILIN2 | PW |  |  | |
| **Q96CU9** | FXRD1_HUMAN | FAD-dependent oxidoreductase | FOXRED1 |  |  | IF | |
| **I3L401** | I3L401_HUMAN | FCP1 homology domain-containing protein | I3L401 |  | PP |  | |
| **P35555** | FBN1_HUMAN | Fibrillin-1 | FBN1 | PW |  |  | |
| **Q75N90** | FBN3_HUMAN | Fibrillin-3 | FBN3 |  |  | IF | |
| **P49915** | GUAA_HUMAN | GMP synthase | GMPS |  | PP |  | |
| **A0A140TA62** | A0A140TA62_HUMAN | IF rod domain-containing protein | LOC100653049 |  | PP |  | |
| [**Q6P089**](https://www.uniprot.org/uniprotkb/Q6P089/entry) | Q6P089_HUMAN | [IGH@ protein](mailto:IGH@%20protein) | [IGH@](mailto:IGH@) | PW | PP |  | |
| **Q0KKI6** | Q0KKI6_HUMAN | Immunoblobulin light chain (Fragment) | Q0KKI6 | PW |  |  | |
| **P0DOX2** | IGA2_HUMAN | Immunoglobulin alpha-2 heavy chain | IgH |  | PP |  | |
| **Q9NPP6** | Q9NPP6_HUMAN | Immunoglobulin heavy chain variant | IGH | PW | PP |  | |
| **P01876** | IGHA1_HUMAN | Immunoglobulin heavy constant alpha 1 | IGHA1 | PW | PP |  | |
| **P01877** | IGHA2_HUMAN | Immunoglobulin heavy constant alpha 2 | IGHA2 | PW | PP |  | |
| **P13645** | K1C10_HUMAN | Keratin, type I cytoskeletal 10 | KRT10 | PW |  |  | |
| **Q9C075** | K1C23_HUMAN | Keratin, type I cytoskeletal 23 | KRT23 |  | PP |  | |
| **P61626** | LYSC_HUMAN | Lysozyme C | LYZ |  | PP | IF | |
| **Q8N119** | MMP21_HUMAN | Matrix metalloproteinase-21 | MMP21 |  |  | IF | |
| **Q9HC84** | MUC5B_HUMAN | Mucin-5B | MUC5B | PW |  |  | |
| **P59665** | DEF1_HUMAN | Neutrophil defensin 1 | DEFA1 | PW |  |  | |
| **P59666** | DEF3_HUMAN | Neutrophil defensin 3 | DEFA3 | PW |  |  | |
| **P14543** | NID1_HUMAN | Nidogen-1 | NID1 |  |  | IF | |
| **Q8N323** | NXPE1_HUMAN | NXPE family member 1 | NXPE1 |  | PP |  | |
| **P04746** | AMYP_HUMAN | Pancreatic alpha-amylase | AMY2A | PW | PP | IF | |
| **Q92626** | PXDN_HUMAN | Peroxidasin homolog | PXDN | PW |  |  | |
| **P14555** | PA2GA_HUMAN | Phospholipase A2 (Phospholipase A2, membrane associated) | PLA2G2A |  |  | IF | |
| **P01833** | PIGR_HUMAN | Polymeric immunoglobulin receptor | PIGR |  |  | IF | |
| **P49792** | RBP2_HUMAN | RanBP2-type domain-containing protein | RANBP2 |  |  | IF | |
| **Q8WY24** | Q8WY24_HUMAN | SNC66 protein | SNC66 | PW | PP |  | |
| **Q9UP60** | Q9UP60_HUMAN | SNC73 protein | SNC73 | PW |  |  | |
| **Q92673** | SORL_HUMAN | Sortilin-related receptor | SORL1 |  | PP |  | |
| **O43897** | TLL1_HUMAN | Tolloid-like protein 1 | TLL1 |  |  | IF | |
| **Q9NVV0** | TM38B_HUMAN | Trimeric intracellular cation channel family protein | TMEM38B |  |  | IF | |
| **Q96DK0** | Q96DK0_HUMAN | Tro alpha1 H,myeloma - cDNA FLJ41981 | Q96DK0 | PW |  |  | |
| **Q6ZVX0** | Q6ZVX0_HUMAN | Tro alpha1 H,myeloma - cDNA FLJ25298I | Q6ZVX0 |  | PP |  | |
| **Q9Y5K8** | VATD_HUMAN | V-type proton ATPase subunit D | ATP6V1D |  |  | IF | |
| **Q8N972** | ZN709_HUMAN | Zinc finger protein 709 | ZNF709 |  | PP |  | |
